# Association between superior longitudinal fasciculus, motor recovery, and motor outcome after stroke: a cohort study

**DOI:** 10.3389/fneur.2023.1157625

**Published:** 2023-07-14

**Authors:** Thomas Jacquemont, Romain Valabregue, Lina Daghsen, Eric Moulton, Chiara Zavanone, Jean Charles Lamy, Charlotte Rosso

**Affiliations:** ^1^Inserm U 1127, CNRS UMR 7225, Sorbonne Université, Institut du Cerveau et de la Moelle épinière, ICM, Paris, France; ^2^STARE Team, iCRIN, Institut du Cerveau et de la Moelle épinière, ICM, Paris, France; ^3^Centre de Neuro-Imagerie de Recherche, CENIR, ICM, Paris, France; ^4^APHP-Service de Soins de Suite et Réeducation, Hôpital Pitié-Salpêtrière, Paris, France; ^5^APHP-Urgences Cérébro-Vasculaires, Hôpital Pitié-Salpêtrière, Paris, France

**Keywords:** stroke, motor recovery, motor outcome, superior longitudinal fasciculus, fractional anisotropy, diffusion tensor

## Abstract

**Introduction:**

Parieto-frontal interactions are mediated by the superior longitudinal fasciculus (SLF) and are crucial to integrate visuomotor information and mediate fine motor control. In this study, we aimed to characterize the relation of white matter integrity of both parts of the SLF (SLF I and SLF II) to both motor outcome and recovery and its evolution over time in stroke patients with upper limb motor deficits.

**Materials and methods:**

Fractional anisotropy (FA) values over the SLF I, SLF II, and corticospinal tract (CST) and upper limb motor performance evaluated by both the upper limb Fugl-Meyer Assessment score and maximum grip strength were measured for 16 patients at 3 weeks, 6 weeks, and 12 weeks poststroke. FA changes were assessed over time using repeated-measures Friedman ANOVA, and correlations between motor recovery, motor outcome at 12 weeks, and FA values in the CST, SLF I, and SLF II at 3 weeks were performed using Spearman's rank-order correlation.

**Results:**

FA values in the affected hemisphere's SLF I and SLF II at 3 weeks correlated with motor recovery at 12 weeks when assessed by the Fugl-Meyer Assessment for upper limb extremity (rho: 0.502, p: 0.04 and rho: 0.510, p: 0.04, respectively) but not when assessed by grip strength. FA values in the SLF I and SLF II were not correlated with motor outcomes. FA values in the SLF II in the affected hemisphere changed significantly over time (p: 0.016).

**Conclusion:**

Both SLF I and SLF II appeared to participate in poststroke motor recovery of complex movements but not in the motor outcome. These results argue that visually/spatially oriented motor tasks as well as more complex motor tasks using parietal associative areas should be used for poststroke rehabilitation strategies.

## 1. Introduction

Parieto-frontal interactions are crucial in healthy subjects to transfer information for movement planning as well as to integrate visuomotor information and to mediate fine motor control (1). These processes are necessary for the correct, goal-oriented movement of the upper limb. It has also been shown that the microstructural integrity of the white matter bundles within the parieto-frontal network plays a prominent role when action reprogramming, required for the on-line control of reaching movements, is interfered (2). The anatomical substrate of these parieto-frontal interactions is mainly the superior longitudinal fasciculus with its two parts (SLF I and II) (3). SLF I links the superior parietal lobule and the medial parietal cortex (which are part of the posterior parietal cortex) with secondary motor areas, such as the premotor area (4, 5). SLF II travels ventrally and laterally to SLF I and connects the inferior parietal lobule to more anterior areas in the frontal lobe (superior frontal gyrus) (4).

In stroke patients, residual motor function of the upper limb is usually explained by corticospinal tract integrity (CST), but not sufficiently. Few studies have investigated how parieto-frontal connections contribute to residual motor function in cross-sectional studies recruiting patients in the chronic phase (6–8). Schulz et al. (6) showed that in chronic patients the upper limb motor outcome was partly explained by the integrity of the parieto-frontal projections belonging to the SLF. In the study by Moulton et al. (7), regions associated with poor motor prognosis included the posterior parietal cortex, where the SLF I originates. Using resting-state functional MRI, Hordacre et al. (8) demonstrated that severely impaired patients had greater performance when functional connectivity in the ipsilesional parieto-frontal network was higher. Taken together, the parieto-frontal network and its anatomical substrates (the SLF I and II) may appear to be a behaviorally relevant neural mechanism that improves upper limb motor performance. However, if the SLF I and II explained part of the variance of the motor outcome, motor recovery (i.e., the gain of function from rehabilitation therapy) was not investigated in these studies.

Therefore, our aim was first to confirm the correlation between SLF diffusion properties and motor recovery and outcome over a 3-month period. Second, we aimed to characterize the diffusion properties (measuring the microstructure integrity of the tract by diffusion tensor imaging) of SLFs I and II in the affected and unaffected hemispheres of stroke patients and their dynamics over time.

To that end, we used the data of a longitudinal cohort study, which aimed to find biomarkers to predict motor recovery by means of repetitive assessments using diffusion tensor imaging and clinical evaluation.

## 2. Materials and methods

### 2.1. Patients

A total of 22 patients were recruited from the stroke unit and the neurorehabilitation department of La Pitié-Salpêtrière Hospital (November 2018 to December 2020) to participate in a study that aimed to find biomarkers to predict motor recovery (ClinicalTrials.gov Identifier: NCT03739892).

The inclusion criteria were as follows: (i) first-ever stroke event (ischemic or hemorrhagic); (ii) impaired upper limb function defined on the Fugl-Meyer Assessment score; and (iii) aged ≥ 18 years old. The exclusion criteria were as follows: (i) suffering from a life-threatening disease during the follow-up period; (ii) contraindications to MRI; (iii) severe aphasia hampering the ability of the patients to follow a rehabilitation program; and (iv) aged > 85 years old.

This study was conducted according to established good clinical practice guidelines and was approved by the local ethical committee (CPP Sud Mediterranée III). Written informed consent from each participant or from a legal proxy/family member was obtained. For this specific substudy, only patients with ischemic stroke were selected, for a total of 16 patients. The presence of fresh blood in the parenchymal hematoma at the subacute stage creates artifacts that can result in lower diffusion values in the affected area, which may not accurately reflect the underlying tissue integrity (9).

The cohort consisted of a longitudinal single-center cohort involving patients who were recruited 3 weeks after a stroke to attend a 6-week standardized rehabilitation program. The 6-week rehabilitation program consisted of daily sessions of rehabilitation combining physical therapy and occupational therapy. In addition, the subjects received daily individual functional rehabilitation for the upper limb using a virtual reality device to increase rehabilitation doses. The follow-up consisted of clinical assessments and clinical imaging, which were performed at inclusion in the study (V1, 3 weeks), 6 weeks from inclusion (V2), and 12 weeks from inclusion (V3).

Upper limb motor performance was evaluated with the Fugl-Meyer Assessment score and maximum grip strength. In this study, we excluded the reflex items of the Fugl-Meyer Assessment score for a maximum score of 60 points (10, 11). For both hands, the maximum grip strength was measured three times and averaged. The grip strength ratio was computed by dividing the score of the affected hand by that of the unaffected hand.

### 2.2. MRI

#### 2.2.1. MRI acquisition

We acquired diffusion with multiband EPI (12), with an isotropic voxel of 1.64 mm, a TR of 4.9 s, a TE of 77 ms, a multiband factor of 2, and a 7/8 partial Fourier. We acquired 7 *b* = 0 s/mm^2^ volumes and 5 different shells: 46, 29, 16, 7, and 3 directions for b-values of 3,000, 2,000, 1,000, 700, and 300 s/mm^2^, respectively. After each series, a volume without diffusion was acquired in the opposite phase direction.

#### 2.2.2. Preprocessing

The data were first denoised, and Gibbs artifacts were removed with the MRtrix 3 software (13). The *b* = 0 volumes were then extracted and combined with the opposite phase direction volumes to perform EPI distortion correction with the topup tool from FSL (5.0) (14). We then corrected the data for motion and eddy current distortions with an eddy tool from FSL (15). Fractional anisotropy (FA) maps were generated using the diffusion tensor model.

#### 2.2.3. Data computation

Stroke lesions were manually outlined by a neurologist on the FLAIR images. For each tract of interest (CST, SLF I, and II), template tracts were obtained from a healthy whole-brain tractography using MRtrix3 [details in Moulton et al. (16) and Supplementary material]. Individual FA maps were registered to the healthy whole-brain tractography space first linearly using the FSL flirt tool with 6 degrees of freedom and then non-linearly using the FSL-fnirt tool with 12 degrees of freedom. During the registration, the lesioned voxels were masked to avoid distortions due to the lesion. We then extracted density-weighted FA values from the affected and unaffected entire fasciculi (17–21).

### 2.3. Statistics

All variables were tested using the Shapiro–Wilk normality test. As normality was not achieved for all variables, we used non-parametric tests for the statistical analysis. The descriptive statistics were the median and interquartile range (IQR). Missing data were not imputed.

Outcome was defined as the upper limb Fugl-Meyer Assessment score or grip strength ratio at V3. Recovery was defined as the difference in upper limb Fugl-Meyer Assessment score (or grip strength ratio) between V3 and V1. Correlations between motor recovery and motor outcome at V3 and FA values in the CST, SLF I, and SLF II at V1 were performed using Spearman's rank-order correlation. Multiple regression models using a stepwise approach were used to explain motor recovery with the difference in Fugl-Meyer Assessment scores between V3 and V1 as the dependent variable and the FA values of the CST, SLF I, and SLF II, as well as the initial Fugl-Meyer Assessment score and age, as the independent variables.

Comparisons of FA values in the tracts of interest (SLF I, SLF II, and CST) between the affected and unaffected hemispheres were performed using the Wilcoxon tests. FA value changes over time were assessed using repeated-measures Friedman ANOVA, and *post-hoc* tests were performed using a Wilcoxon test. We computed axial, radial, and mean diffusivity values (AD, RD, and MD) and investigated their changes over time to highlight the possible mechanisms of FA changes (Supplementary material and Section 3). All tests were corrected for multiple comparisons using the false discovery rate. Statistical analyses were performed using MedCalc (version 12.5.0, Belgium, 2013), and a *p*-value of < 0.05 was considered statistically significant.

## 3. Results

### 3.1. Patients

**Figure 1:**
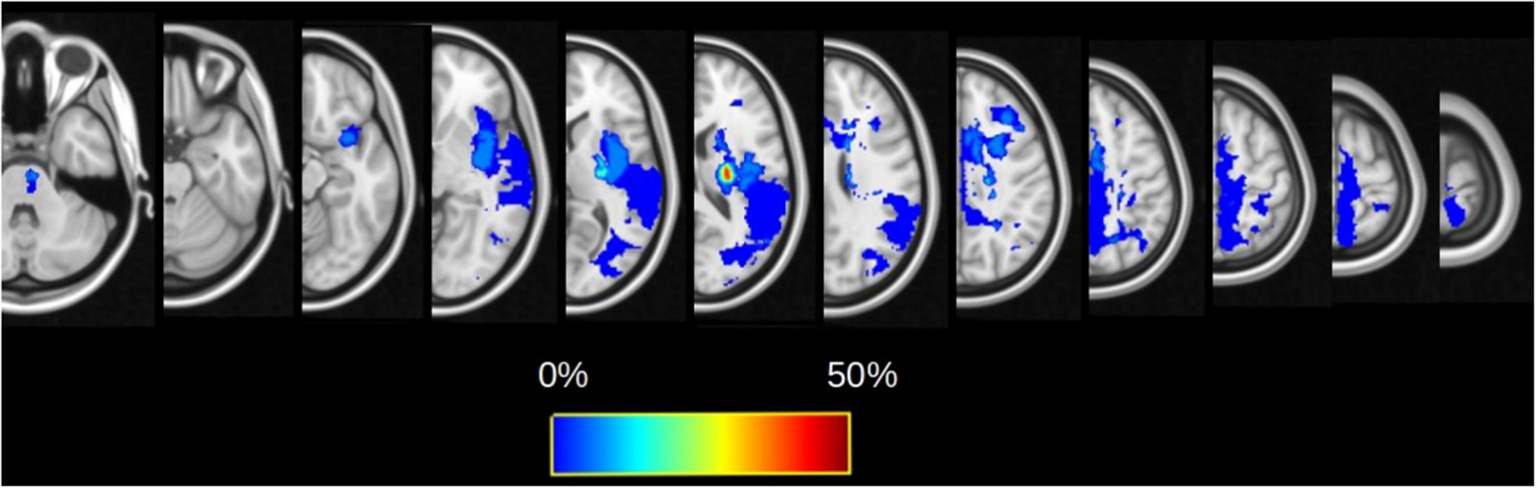
Lesion probability maps for the whole cohort overlaid on the FMRIB58_FA template. The color map reflects the percentage of patients for which the voxel is lesioned by the infarct.

**Figure 2:**
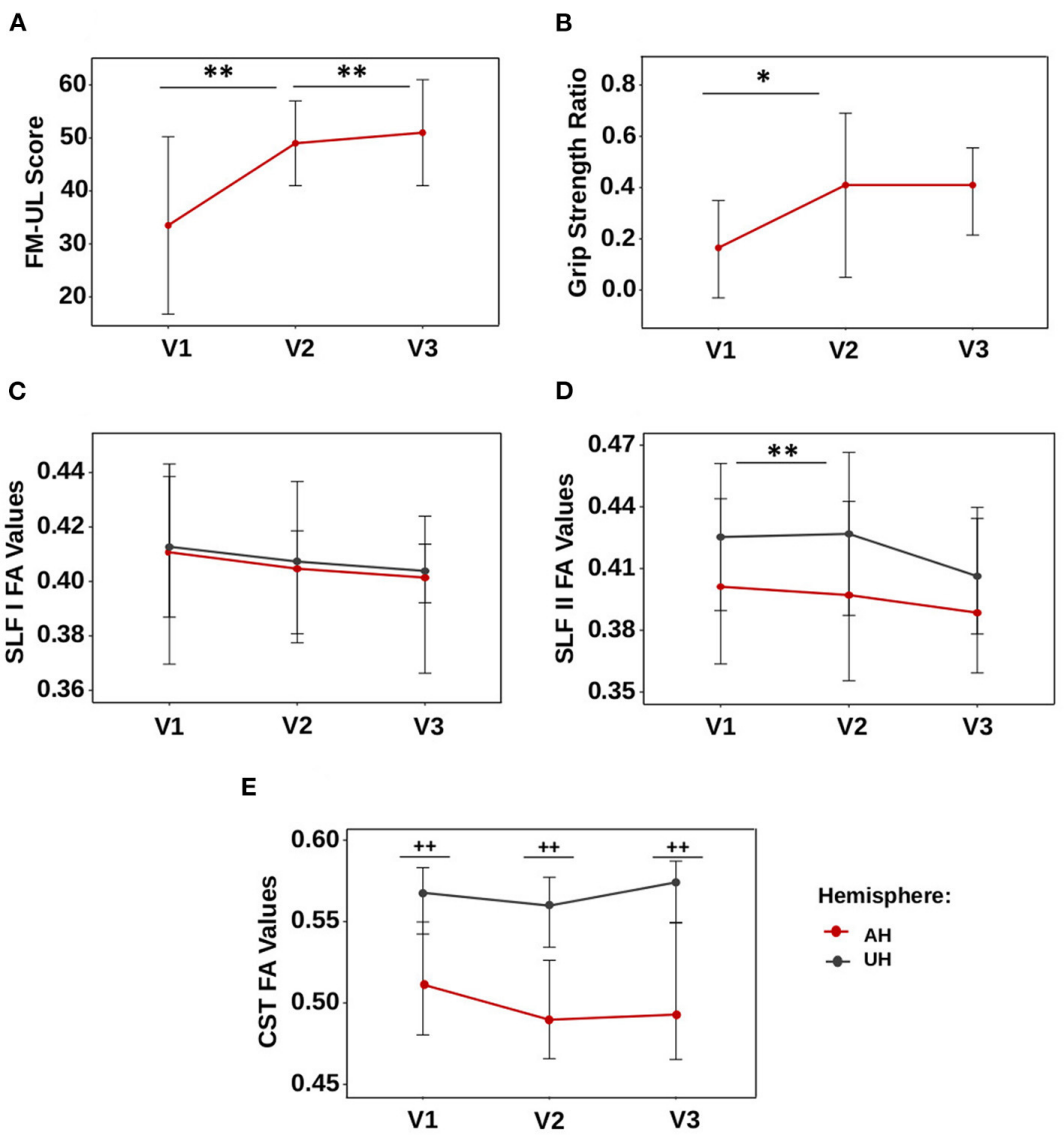
Dynamics of motor scores and FA values. **(A)** Fugl-Meyer assessment, **(B)** GSratio, **(C)** SLF I, **(D)** SLF II, and **(E)** CST. Values are represented by the median, and bars display the interquartile range. *p-value < 0.05, **p-value < 0.01 for the comparison between visits. ^++^*p*-value < 0.01 for comparison between the affected and unaffected hemispheres.

A total of 16 patients (11 men, 68.8%) were analyzed. The median age was 63.5 (57.7–70.2) years. Most of the patients had right-sided lesions (*n* = 10, 62.5%). Lesion locations were the following: 62.5% (*n* = 10) subcortical stroke, 25% (*n* = 4) cortical stroke, and 12.5% (*n* = 2) cortico-subcortical stroke. The lesion probability map is presented in Figure 1, and the highest incidence was located in the internal capsule. The CST overlapped with the ischemic lesion mask in 100% of the patients (*n* = 16), the SLF I in 31.3% (*n* = 5), and the SLF II in 50% (*n* = 8). Clinical scores and FA values are presented in Table 1. Regarding motor recovery measured by the upper limb Fugl-Meyer Assessment, patients improved significantly {[F(3,16): 28.7; *p* < 0.0001, with significant improvement for V1-V2 and V2-V3 with *p* < 0.001 and p: 0.02, respectively; Figure 2A]}. The grip strength ratio also improved {[F(3,15): 13.8, p: 0.001], with significant improvement for V1-V2 with p: 0.02 and stability for V2-V3 with p: 0.20, Figure 2B}.

**Table 1:** Patient characteristics.

	**V1**	**V2**	**V3**
Time poststroke onset (days)	21 (13–31) (9–61)	61 (54.5–76) (51–100)	107 (101–121) (93–150)
Upper limb Fugl-Meyer Assessment	33.5 (25–42) (2–46)	49 (46–54) (32–56)	51 (46–56) (32–58)
Grip strength ratio	0.17 (0.11–0.33) (0–1.11)	0.41 (0.25–0.73) (0.09–1.06)	0.41 (0.35–0.74) (0.11–1.25)
CST affected hemisphere (FA values)	0.511 (0.481–0.528) (0.422–0.600)	0.490 (0.474–0.512) (0.419–0.578)	0.493 (0.462–0.513) (0.415–0.551)
CST unaffected hemisphere (FA values)	0.568 (0.556–0.596) (0.461–0.617)	0.560 (0.550–0.598) (0.458–0.613)	0.574 (0.560–0.589) (0.461–0.613)
SLF I affected hemisphere (FA values)	0.411 (0.386–0.432) (0.137–0.467)	0.405 (0.384–0.429) (0.258–0.467)	0.401 (0.366–0.415) (0.140–0.462)
SLF I unaffected hemisphere (FA values)	0.413 (0.386–0.437) (0.338–0.492)	0.407 (0.387–0.441) (0.339–0.488)	0.404 (0.381–0.449) (0.331–0.481)
SLF II affected hemisphere (FA values)	0.401 (0.386–0.430) (0.329–0.454)	0.397 (0.381–0.431) (0.324–0.453)	0.389 (0.373–0.409) (0.284–0.449)
SLF II unaffected hemisphere (FA values)	0.425 (0.402–0.438) (0.366–0.455)	0.427 (0.397–0.437) (0.368–0.452)	0.406 (0.399–0.427) (0.363–0.460)

Values are median (interquartile range) and (minimum–maximum).

### 3.2. Correlation between SLF FA values at 3 weeks with motor recovery and motor outcome at 3 months

FA values of the affected hemisphere's CST were not correlated with FA values in SLF I (V1, p: 0.46; V2, p: 0.53; V3, p: 0.86) or SLF II (V1, p: 0.41; V2, p: 0.83; V3, p: 0.72). FA values of the SLF I and SLF II were also not correlated (V1, p: 0.48; V2, p: 0.68; V3, p: 0.72).

**Figure 3:**
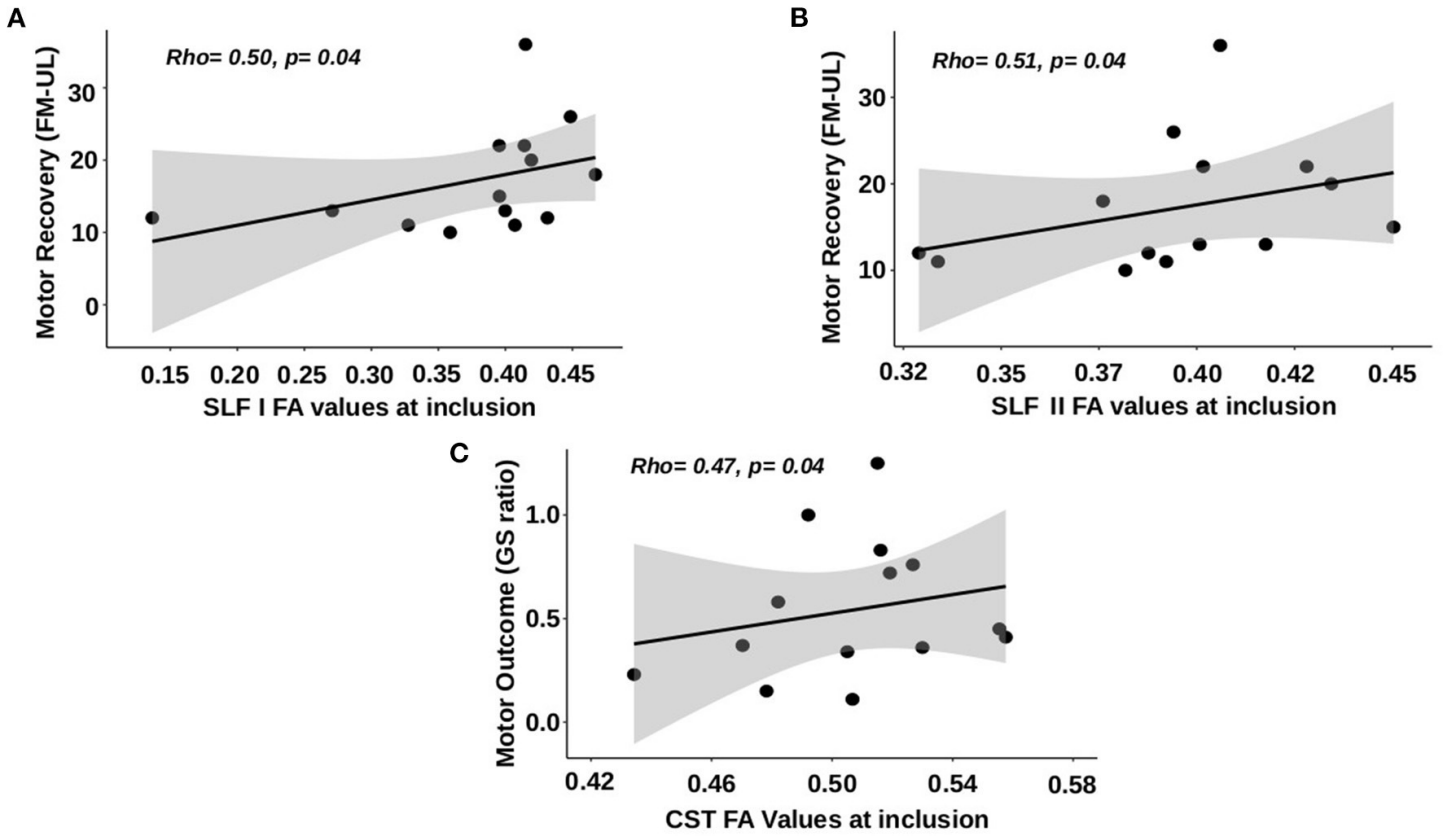
Correlations between FA values at V1 and motor recovery and outcome at 3 months. **(A)** SLF I FA values vs. motor recovery using the upper limb Fugl-Meyer Assessment (FM-UL). **(B)** SLF II FA values vs. motor recovery using FM-UL. **(C)** CST FA values vs. motor outcome using grip strength ratio.

FA values in the SLF I of the affected hemisphere at V1 were correlated with motor recovery assessed by the upper limb Fugl-Meyer Assessment between V1 and V3 (rho: 0.502, 95% CI: 0.007; 0.799, p: 0.04) but not when assessed by the grip strength ratio (p: 0.50). The better the integrity was in the SLF I (high FA values), the better the patients improved (Figure 3A). FA values in the affected hemisphere's SLF II also had a positive correlation with motor recovery assessed by the upper limb Fugl-Meyer Assessment between V1 and V3 (rho: 0.510, 95% CI: 0.197; 0.803 p: 0.04, Figure 3B) but not assessed by the grip strength ratio (p: 0.72). FA values in the affected hemisphere's SFL I and SFL II were neither correlated with the motor outcome at V3 assessed by the upper limb Fugl-Meyer Assessment nor with the grip strength ratio (p: 0.25 and p: 0.81 for the upper limb Fugl-Meyer Assessment; p: 0.16 and p: 0.86 for grip strength ratio). The multiple regression model performed to explain motor recovery retained two independent variables [F(2,13): 24.9, *p* < 0.001], namely, (i) the initial Fugl-Meyer Assessment score and (ii) the FA values in the SLF I. The proportion of variance explained by these two variables was 78.7% (*p* < 0.001). Age (p: 0.26), FA values in the CST (p: 0.36), and SLF II (p: 0.07) were not retained in the models as independent variables. Statistics from the stepwise multiple linear regression models are summarized in Supplementary Table 2.

FA values in the CST of the affected hemisphere were not correlated with motor recovery assessed by the upper limb Fugl-Meyer Assessment (p: 0.67), but there was a tendency for the correlation with the motor recovery assessed by the grip strength ratio between V1 and V3 (rho: 0.485, 95% CI: −0.036; 0.799, p: 0.06). FA values in the affected hemisphere's CST were correlated with the motor outcome assessed by grip strength ratio at V3 (rho = 0.515, 95% CI: 0.067; 0.843; p: 0.04, Figure 3C) but not upper limb Fugl-Meyer Assessment (p: 0.41). The better the integrity was in the CST (high FA values), the better the patient's outcome (high grip strength ratio at V3).

The results of all Spearman's rank-order correlations performed are summarized in Supplementary Table 3.

### 3.3. Characterization of SLF I and II FA values during motor recovery

FA values in the SLF I and SLF II in the affected hemisphere changed significantly over time [F(3,12): 7.16, p: 0.027 and F(3,12): 8.16, p: 0.016, respectively, Figures 2C, D], whereas these remained stable in the unaffected hemisphere (p: 0.49 and p: 0.10, respectively). FA values in the SLF II of the affected hemisphere decreased between V1 and V2 (p: 0.004) but not between V2 and V3 (p: 0.52). FA values in the affected hemisphere's SLF I were not significantly different after correction for multiple comparisons (p: 0.07 for V1-V2; p: 0.62 for V2-V3).

FA values in the SLF I and SLF II were similar in the affected hemisphere compared to the unaffected hemisphere at V1 (SLF I, p: 0.09; SLF II, p: 0.26), V2 (SLF I, p: 0.07; SLF II, p: 0.18), and V3 (SLF I, p: 0.08; SLF II, p: 0.11) (Figures 2C, D).

In contrast, FA values in the CST in the affected and unaffected hemispheres remained stable over time (p: 0.27 and p: 0.21, Figure 2E), but these values were significantly lower in the affected hemisphere than in the unaffected hemisphere at V1, V2, and V3 (*p*-values were < 0.004 at V1 and V2 and < 0.006 at V3).

## 4. Discussion

This study is the first longitudinal study using diffusion tensor imaging to investigate the role of the SLF in motor outcomes and recovery at 3 months. We used diffusion tensor imaging to investigate the relationship between the SLF FA values and both motor recovery and motor outcome at 3 months and to assess the evolution of SLF FA values during recovery in ischemic strokes. Interestingly, FA values in SLF I and II were correlated with motor recovery but not outcome assessed by the upper limb Fugl-Meyer Assessment, whereas CST integrity in the affected hemisphere was correlated with motor outcome assessed by the grip strength ratio (and tended to be correlated with motor recovery with the grip strength ratio and not the Fugl-Meyer Assessment). This suggested that SLFs I and II contributed more to motor improvements of more complex movements and motor control than pure strength of the hand.

### 4.1. Correlation between SLF FA values and motor recovery and motor outcome at 3 months

SLF I originates from the superior parietal lobe and projects ventrally along the cingulate gyrus toward the superior frontal gyrus, terminating within the supplementary motor and premotor areas (22). This tract is known to play an important role in gathering proprioception information and motor movement initiation (5, 23). A recent study using both diffusion tensor imaging and functional imaging showed that, among the complex network connected by the different parts of the SLF, the SLF I bundle is the main tract associated with motor function using spatial information (1). Our results suggest that SLF I might play a role in motor recovery poststroke. A hypothetical mechanism (among others) may involve using information from non-motor multiple regression models (especially spatial information). Parieto-frontal interactions are crucial in healthy subjects to integrate visuomotor information and to mediate fine motor control during the on-line control of reaching movements (1, 2). Based on this hypothesis, rehabilitation tasks that are oriented toward spatial information, such as “goal-oriented tasks,” may help facilitate motor recovery poststroke. This hypothesis could explain why SLF I was correlated with motor recovery assessed by the Fugl-Meyer Assessment and not by the grip strength ratio, which is a measurement of pure muscle strength. Indeed, after a stroke, there is not only a general loss of force but also the occurrence of synergy, leading to non-optimal patterns of movement. Many daily tasks consist of squeezing objects and involve coordination and precision, such as picking up small objects, closing bottle caps, and other similar manipulations. It is plausible, given the role of the SLF in the healthy brain, that SLF integrity is correlated (i) first with the Fugl-Meyer Assessment score, which is a motor score centered on synergistic movements (and not grip strength) and (ii) then with recovery (i.e., the gain of function during rehabilitation) and not the outcome. Indeed, in the healthy brain, the SLF lays connections between the frontal lobe and the posterior parietal cortex, which is known to subserve sensorimotor integration, including visuomotor mapping, on-line adaptation, and error monitoring, necessary for task completion (24). All these processes are required for motor learning in the healthy brain and so are they in the recovery process when rehabilitation strategies rely on repetitive practice (25).

SLF II's mechanism for motor recovery appeared to be similar. Indeed, SLF II FA values displayed a significant correlation with motor recovery at 3 months. SLF II principally originates around the angular gyrus and targets both dorsolateral frontal brain regions and the ventral premotor cortex (4–6, 26, 27). If SLF I is implied only in spatially associated motor function, the SLF II role seems to be mixed and is involved in both spatial and non-spatial motor function (1). This is consistent with Abela et al. (28), who reported that gray matter atrophy in the angular gyrus was associated with less favorable recovery after ischemic stroke (28).

In our study, CST FA values correlated with the motor outcome using grip strength ratio (but not upper limb Fugl-Meyer) and tended to correlate with motor recovery (29, 30). However, even if CST white matter integrity during a stroke is known to be a promising biomarker for the prediction of the motor outcome, to date, studies' results are inconsistent, and several of them failed to find such a correlation with motor recovery, i.e., gain from therapy (17, 31–36). An explanation for the correlation of the CST with grip strength and not the Fugl-Meyer Assessment might be related to the motor control of proximal upper limb muscles vs. digits (25). Lawrence and Kuypers (37) described that monkeys with bilateral corticospinal tract lesions lost their ability to individuate the digits, a state that remained for months. In contrast, the proximal limb movements returned, although impaired (37). This experiment stressed that the CST is more dedicated to digit control than proximal arm control.

The discrepancies between CST integrity and motor recovery/outcome can also be explained by the heterogeneity in diffusion tensor imaging studies of patient populations and methodologies. One important source of discrepancy is the methodology used to assess the CST white matter integrity (i.e., which diffusion tensor imaging metrics and which methods to use for CST tract reconstruction) (18). Li et al. (21) reported, in a tractometry-based study, that, if mean diffusivity, axial diffusivity, and radial diffusivity correlated with the modified Rankin scale at 3 months, FA failed to reach statistical significance.

### 4.2. Characterization of SLF I and II FA values during motor recovery

To our knowledge, our study allowed the first longitudinal characterization of SLF FA values after a stroke in the subacute phase. We found that SLF II diffusion properties in the affected hemisphere significantly changed over time from the early subacute phase to 3 months. The results of the change in FA in the SLF I were not significant after correction for multiple comparisons. SLF II displayed a decrease in FA values, which could suggest Wallerian degeneration. Filatova et al. (38) observed SLF FA values in chronic stroke patients (more than 6 months poststroke) and reported a significant difference in FA asymmetry in the SLF between hemispheres compared to healthy subjects. In our study, SLF II changes were only significant between V1 and V2 but not between V2 and V3. This decrease in FA values in the SLF over time reflects secondary axonal damage. Among the possible mechanisms, Wallerian degeneration is probably the main mechanism at play (as reported in the CST) (31, 39, 40).

### 4.3. Study limitations

Our study has some limitations that need to be identified. First, the main limitation of our study is the small number of patients (*n* = 16), probably explaining the lack of power of some analyses, especially for a diffusion tensor imaging study. Indeed, diffusion tensor imaging is a noise-sensitive and artifact-prone sequence, and a lack of power in our study is possible (41). More generally, further studies are needed to confirm the findings of our study and illuminate the shadow areas. The second limitation relies on the absence of an age-matched cohort of healthy subjects. It would have allowed us to provide data for the comparison of contralesional values for FA measurements. However, the status of the contralesional hemisphere was beyond the scope of our study, which focused on the correlation between poststroke motor recovery and the microstructural integrity of SLF and CST in the ipsilesional hemisphere. Third, the small sample size of our cohort was also an issue. For example, we could not perform subgroup analyses according to the location of lesions, although these could have an impact on our results. Finally, as in any study, the results depend on the population criteria, though the generalizability of our results might be limited.

## 5. Conclusion

Both SLF I and SLF II appeared to participate in poststroke motor recovery but not in the motor outcome. Together, our results argued that, considering the participation of the SLF in motor recovery, visually/spatially oriented motor tasks, as well as more complex motor tasks using parietal associative areas, should be used for poststroke rehabilitation strategies.

## Data availability statement

The raw data supporting the conclusions of this article will be made available upon reasonable request to the corresponding author and if it does not violate the GDPR and local ethics committee rules.

## Ethics statement

The studies involving human participants were reviewed and approved by CPP Sud Mediterranée III. The patients/participants provided their written informed consent to participate in this study.

## Author contributions

Study conception and design: CR and JL. Data collection: CR, CZ, and LD. Analysis and interpretation of results: CR, TJ, RV, and EM. Draft manuscript preparation: TJ and CR. All authors reviewed the results and approved the final version of the manuscript.

## References

[1] Parlatini V Radua J Dell'Acqua F Leslie A Simmons A Murphy DG. Functional segregation and integration within fronto-parietal networks. NeuroImage. (2017) 146:367–75. 10.1016/j.neuroimage.2016.08.031 27639357 PMC5312783

[2] Rodríguez-Herreros B Amengual JL Gurtubay-Antolín A Richter L Jauer P Erdmann C. Microstructure of the superior longitudinal fasciculus predicts stimulation-induced interference with on-line motor control. Neuroimage. (2015) 120:254–65. 10.1016/j.neuroimage.2015.06.070 26143205

[3] Thiebaut de Schotten M Dell'Acqua F Valabregue R Catani M. Monkey to human comparative anatomy of the frontal lobe association tracts. Cortex. (2012) 48:82–96. 10.1016/j.cortex.2011.10.001 22088488

[4] Petrides M Pandya DN. Projections to the frontal cortex from the posterior parietal region in the rhesus monkey. J Comp Neurol. (1984) 228:105–16. 10.1002/cne.902280110 6480903

[5] Schmahmann JD Smith EE Eichler FS Filley CM. Cerebral white matter. Ann N Y Acad Sci. (2008) 1142:266–309. 10.1196/annals.1444.017 18990132 PMC3753195

[6] Schulz R Koch P Zimerman M Wessel M Bönstrup M Thomalla G. Parietofrontal motor pathways and their association with motor function after stroke. Brain. (2015) 138:1949–60. 10.1093/brain/awv100 25935722

[7] Moulton E Magno S Valabregue R Amor-Sahli M Pires C Lehéricy S. Acute diffusivity biomarkers for prediction of motor and language outcome in mild-to-severe stroke patients. Stroke. (2019) 50:2050–6. 10.1161/STROKEAHA.119.024946 31272324

[8] Hordacre B Lotze M Jenkinson M Lazari A Barras CD Boyd L. Fronto-parietal involvement in chronic stroke motor performance when corticospinal tract integrity is compromised. NeuroImage Clin. (2021) 29:102558. 10.1016/j.nicl.2021.102558 33513561 PMC7841401

[9] Puig J Blasco G Terceño M Daunis-I-Estadella P Schlaug G Hernandez-Perez M. Predicting motor outcome in acute intracerebral hemorrhage. Am J Neuroradiol. (2019) 40:769–75. 10.3174/ajnr.A6038 31000524 PMC7053898

[10] Gladstone DJ Danells CJ Black SE. The fugl-meyer assessment of motor recovery after stroke: a critical review of its measurement properties. Neurorehabil Neural Repair. (2002) 16:232–40. 10.1177/154596802401105171 12234086

[11] Hsueh I-P Hsu M-J Sheu C-F Lee S Hsieh C-L Lin J-H. Psychometric comparisons of 2 versions of the Fugl-Meyer motor scale and 2 versions of the stroke rehabilitation assessment of movement. Neurorehabil Neural Repair. (2008) 22:737–44. 10.1177/1545968308315999 18645189

[12] Auerbach EJ Xu J Yacoub E Moeller S Ugurbil K. Multiband accelerated spin-echo echo planar imaging with reduced peak RF power using time-shifted RF pulses. Magn Reson Med. (2013) 69:1261–7. 10.1002/mrm.24719 23468087 PMC3769699

[13] Tournier JD Smith R Raffelt D Tabbara R Dhollander T Pietsch M. MRtrix3: a fast, flexible and open software framework for medical image processing and visualization. NeuroImage. (2019) 202:116137. 10.1016/j.neuroimage.2019.116137 31473352

[14] Andersson JLR Skare S Ashburner J. How to correct susceptibility distortions in spin-echo echo-planar images: application to diffusion tensor imaging. Neuroimage. (2003) 20:870–88. 10.1016/S1053-8119(03)00336-7 14568458

[15] Andersson JLR Sotiropoulos SN. An integrated approach to correction for off-resonance effects and subject movement in diffusion MR imaging. Neuroimage. (2016) 125:1063–78. 10.1016/j.neuroimage.2015.10.019 26481672 PMC4692656

[16] Moulton E Valabregue R Díaz B Kemlin C Leder S Lehéricy S. Comparison of spatial normalization strategies of diffusion MRI data for studying motor outcome in subacute-chronic and acute stroke. Neuroimage. (2018) 183:186–99. 10.1016/j.neuroimage.2018.08.002 30086410

[17] Groisser BN Copen WA Singhal AB Hirai KK Schaechter JD. Corticospinal tract diffusion abnormalities early after stroke predict motor outcome. Neurorehabil Neural Repair. (2014) 28:751–60. 10.1177/1545968314521896 24519021 PMC4128905

[18] Feldman SJ Boyd LA Neva JL Peters S Hayward KS. Extraction of corticospinal tract microstructural properties in chronic stroke. J Neurosci Methods. (2018) 301:34–42. 10.1016/j.jneumeth.2018.03.001 29522781

[19] Peters DM Fridriksson J Richardson JD Stewart JC Rorden C Bonilha L. Upper and lower limb motor function correlates with ipsilesional corticospinal tract and red nucleus structural integrity in chronic stroke: a cross-sectional, ROI-based MRI study. Behav Neurol. (2021) 2021:3010555. 10.1155/2021/3010555 34804258 PMC8601844

[20] Lee J Chang WH Kim Y-H. Relationship between the Corticospinal and Corticocerebellar tracts and their role in upper extremity motor recovery in stroke patients. J Pers Med. (2021) 11:1162. 10.3390/jpm11111162 34834514 PMC8620974

[21] Li Y Yan S Zhang G Shen N Wu D Lu J. Tractometry-based estimation of corticospinal tract injury to assess initial impairment and predict functional outcomes in ischemic stroke patients. J Magn Reson Imaging. (2022) 55:1171–80. 10.1002/jmri.27911 34487595

[22] Jang SH Hong JH. The anatomical characteristics of superior longitudinal fasciculus I in human brain: Diffusion tensor tractography study. Neurosci Lett. (2012) 506:146–8. 10.1016/j.neulet.2011.10.069 22085696

[23] Chang EF Raygor K Berger MS. Contemporary model of language organization: an overview for neurosurgeons. J Neurosurg. (2015) 122:250–61. 10.3171/2014.10.JNS132647 25423277

[24] Blakemore S-J Sirigu A. Action prediction in the cerebellum and in the parietal lobe. Exp Brain Res. (2003) 153:239–45. 10.1007/s00221-003-1597-z 12955381

[25] Krakauer JW. Motor learning: its relevance to stroke recovery and neurorehabilitation ≫:, Curr. Opin Neurol. (2006) 19:84–90. 10.1097/01.wco.0000200544.29915.cc 16415682

[26] Croxson PL Johansen-Berg H Behrens TEJ Robson MD Pinsk MA Gross CG. Quantitative investigation of connections of the prefrontal cortex in the human and macaque using probabilistic diffusion tractography. J Neurosci Off J Soc Neurosci. (2005) 25:8854–66. 10.1523/JNEUROSCI.1311-05.2005 16192375 PMC6725599

[27] Rozzi S Calzavara R Belmalih A Borra E Gregoriou GG Matelli M. Cortical connections of the inferior parietal cortical convexity of the macaque monkey. Cereb Cortex. (2006) 16:1389–417. 10.1093/cercor/bhj076 16306322

[28] Abela E Seiler A Missimer JH Federspiel A Hess CW Sturzenegger M. Grey matter volumetric changes related to recovery from hand paresis after cortical sensorimotor stroke. Brain Struct Funct. (2015) 220:2533–50. 10.1007/s00429-014-0804-y 24906703 PMC4549385

[29] Wen H Alshikho MJ Wang Y Luo X Zafonte R Herbert MR. Correlation of fractional anisotropy with motor recovery in patients with stroke after postacute rehabilitation. Arch Phys Med Rehabil. (2016) 97:1487–95. 10.1016/j.apmr.2016.04.010 27178097 PMC6037410

[30] Kumar P Yadav AK Misra S Kumar A Chakravarty K Prasad K. Prediction of upper extremity motor recovery after subacute intracerebral hemorrhage through diffusion tensor imaging: a systematic review and meta-analysis. Neuroradiology. (2016) 58:1043–50. 10.1007/s00234-016-1718-6 27438802

[31] Puig J Pedraza S Blasco G Daunis-I-Estadella J Prats A Prados F. Wallerian degeneration in the corticospinal tract evaluated by diffusion tensor imaging correlates with motor deficit 30 days after middle cerebral artery ischemic stroke. Am J Neuroradiol. (2010) 31:1324–30. 10.3174/ajnr.A2038 20299434 PMC7965455

[32] Moura LM Luccas R de Paiva JPQ Amaro E Jr Leemans A Leite CDC. Diffusion tensor imaging biomarkers to predict motor outcomes in stroke: a narrative review. Front Neurol. (2019) 10:445. 10.3389/fneur.2019.00445 31156529 PMC6530391

[33] Ma C Liu A Li Z Zhou X Zhou S. Longitudinal study of diffusion tensor imaging properties of affected cortical spinal tracts in acute and chronic hemorrhagic stroke. J Clin Neurosci. (2014) 21:1388–92. 10.1016/j.jocn.2013.11.032 24746110

[34] Kusano Y Seguchi T Horiuchi T Kakizawa Y Kobayashi T Tanaka Y. Prediction of functional outcome in acute cerebral hemorrhage using diffusion tensor imaging at 3T: a prospective study. Am J Neuroradiol. (2009) 30:1561–5. 10.3174/ajnr.A1639 19556354 PMC7051627

[35] Jang SH Lee J Lee MY Park SM Choi WH Do KH. Prediction of motor outcome using remaining corticospinal tract in patients with pontine infarct: diffusion tensor imaging study. Somatosens Mot Res. (2016) 33:99–103. 10.1080/08990220.2016.1194821 27323912

[36] Zolkefley MKI Firwana YMS Hatta HZM Rowbin C Nassir CMNCM Hanafi MH. An overview of fractional anisotropy as a reliable quantitative measurement for the corticospinal tract (CST) integrity in correlation with a Fugl-Meyer assessment in stroke rehabilitation. J Phys Ther Sci. (2021) 33:75–83. 10.1589/jpts.33.75 33519079 PMC7829559

[37] Lawrence DG Kuypers HGJM. The functional organization of the motor system in the monkey. I. The effects of bilateral pyramidal lesionS. Brain. (1968) 91:1–14. 10.1093/brain/91.1.1 4966862

[38] Filatova OG van Vliet LJ Schouten AC Kwakkel G van der Helm FCT Vos FM. Comparison of multi-tensor diffusion models' performance for white matter integrity estimation in chronic stroke. Front Neurosci. (2018) 12:247. 10.3389/fnins.2018.00247 29740269 PMC5925961

[39] DeVetten G Coutts SB Hill MD Goyal M Eesa M O'Brien B. Acute corticospinal tract Wallerian degeneration is associated with stroke outcome. Stroke. (2010) 41:751–6. 10.1161/STROKEAHA.109.573287 20203322

[40] Thomalla G Glauche V Koch MA Beaulieu C Weiller C Röther J. Diffusion tensor imaging detects early Wallerian degeneration of the pyramidal tract after ischemic stroke. Neuroimage. (2004) 22:1767–74. 10.1016/j.neuroimage.2004.03.041 15275932

[41] Tournier J-D Mori S Leemans A. Diffusion tensor imaging and beyond: diffusion tensor imaging and beyond. Magn Reson Med. (2011) 65:1532–56. 10.1002/mrm.22924 21469191 PMC3366862

