# Supplementary material for: Association between superior longitudinal fasciculus, motor recovery, and motor outcome after stroke: a cohort study
Source: Front Neurol. 2023 Jul 14;14:1157625. doi: 10.3389/fneur.2023.1157625 (PMC10375792; doi:10.3389/fneur.2023.1157625)
Supplement: Supplementary file 1 [file Data_Sheet_1.docx]

**Supplementary Material**

1. MATERIALS AND METHODS

The creation of the tracts templates followed two steps : (1) the creation of an in-house Fiber Orientation Distribution template, and (2) the dissections of the tracts-of-interest.

Creation of in-house study-specific template as described in Moulton et al. 2018 [1]

Data from twenty-four healthy controls (10 females, age=31.7±10.4 years) acquired with a different imaging protocol were used to create the in-house templates for image normalization. Subjects were scanned with a 3T MRI (Siemens, VERIO) with a 32-channel head coil. A multi-shell Diffusion Weighted Imaging (DWI) sequence (3 b-value shells obtained with both posterior to anterior (PA) and anterior to posterior (AP) phase encoding: 60 non-collinear diffusion encoding gradients at b=1500s/mm^2^,30 at b=700s/mm^2^, and 8 at b=300 s/mm^2^, TR=4000 ms, TE=87.8 ms, matrix size=110*110, slice number=66, voxel size=2*2*2mm^3^, acquisition time=16:16 min).

Image processing was the same as the stroke cohort in the current paper, with the exception of FSL’s TOPUP as an additional step to take advantage of the opposite phase-encoded images.

Fiber Orientation Distribution (FOD) volumes were computed by estimating response functions for the grey, white matter, and cerebrospinal tissues for multi-shell multi-tissue constrained spherical deconvolution (MSMT-CSD) using a lmax of 4 [2].

We began by creating a FOD template through iterative non-linear warping and averaging of FOD volumes using MRtrix’s population_template function ([http://www.mrtrix.org](http://www.mrtrix.org/)) [3]. The final resolution of the template was 2x2x2mm^3^.

Tractography with the group template

A tractography analysis was used to virtually dissect white matter fasciculi of interest. We performed whole brain probabilistic tractography on the FOD template using the second-order integration over fiber orientation distribution (iFOD2) algorithm with the following parameters (number of streamlines=100 million, max length=250mm, step size=1mm, max angle=45°). Streamlines were subsequently filtered down to 10 million using the SIFT algorithm [4].

Adhering to the procedures outlined in Rojkova et al. (2016) and Catani and Thiebaut de Schotten (2008), we virtually dissected the (1) the first and second branches of the superior longitudinal fasciculus (SLF) and (2) the corticopinal tract (CST) [5], [6].

**RESULTS**

Axial, radial and mean diffusivity values in the CST and in the two branches of the SLF were calculated. Descriptive statistics are reported in supplementary Table 1.

**Table 1. Description of axial, radial and mean diffusivities in the CST, SLF I and II**

Values are median [interquartile range] and [minimum-maximum].

|  | Ipsilesional hemisphere | | | Controlesional hemisphere | | |
| --- | --- | --- | --- | --- | --- | --- |
|  | V1 | V2 | V3 | V1 | V2 | V3 |
| Axial diffusivity values (*10^-6^) | | | | | | |
| CST | 645  [609-654] [586-716] | 648  [619-661] [587-700] | 665  [631-678] [618-710] | 668  [640-685] [626-720] | 657  [638-685] [609-713] | 677  [638-686] [612-700] |
| SLF I | 676  [656-701] [637-739] | 685  [661-697] [623-740] | 683  [668-689] [644-733] | 686  [668-697] [643-734] | 682  [663-697] [634-727] | 681  [661-695] [629-710] |
| SLF II | 661  [637-687] [629-728] | 665  [644-686] [629-698] | 677  [647-692] [633-792] | 661  [641-683] [623-695] | 664  [636-671] [612-689] | 662  [634-678] [623-698] |
| Radial diffusivity values (*10^-6^) | | | | | | |
| CST | 279  [273-295] [258-319] | 288  [274-307] [262-330] | 286  [277-320] [258-330] | 280  [267-300] [252-325] | 293  [275-312 [256-336] | 290  [271-325] [252-334] |
| SLF I | 390  [384-400] [366-539] | 394  [379-403] [366-482] | 396  [380-412] [370-610] | 383  [378-405] [345-453] | 378  [376-398] [341-460] | 379  [374-427] [344-459] |
| SLF II | 374  [358-398] [341-467] | 382  [356-400] [343-432] | 394  [365-421] [340-542] | 359  [354-392] [334-407] | 359  [355-384] [332-408] | 372  [355-395] [327-406] |
| Mean diffusivity values (*10^-6^) | | | | | | |
| CST | 401  [393-414] [367-429] | 414  [397-421] [380-440] | 417  [406-425] [381-450] | 410  [407-418] [387-434] | 413  [403-427] [384-444] | 417  [402-429] [375-451] |
| SLF I | 487  [478-508] [462-572] | 495  [475-504] [454-556] | 494  [476-505] [464-645] | 479  [468-511] [457-537] | 476  [469-500] [450-546] | 475  [471-511] [453-543] |
| SLF II | 472  [454-489] [440-554] | 473  [452-500] [438-513] | 488  [467-511] [438-625] | 458  [452-489] [433-499] | 461  [449-479] [429-501] | 460  [450-490] [427-499] |

Changes of AD, RD, and MD values were examined using Friedman’s ANOVA. A significant increase in RD values between V1 and V2 was detected in the ipsilesional SLF II, matching the results of fractional anisotropy in this tract.

This is consistent with the fact that fractional anisotropy may be decreased in situations of increased perpendicular (radial) diffusivity [7]. This is also consistent with our hypothesis that FA decrease reflects wallerian degeneration since it has been shown that wallerian degeneration begins with axonal fragmentation (reflected by an reduction of axial diffusivity) whereas at 2 months, an increase in radial diffusivity appear corresponding to myelin degradation [8].

Multiple regression models using a stepwise approach were used to explain motor recovery with the difference of Fugl-Meyer score between V3 and V1 as the dependent variable and with the FA values of the CST, SLF I and II, as well as the initial Fugl-Meyer score and age as the independent variables. The results are reported in supplementary Table 2.

**Table 2. Fugl-Meyer score recovery between V1 and V3 explained with the FA values of the CST, SLF I and II, the initial Fugl-Meyer score at V1 and age using a multiple regression models with a stepwise approach.**

Final multiple regression model’s values are diplayed in bold. Coefficient, standard error et p value for excluded variables were obtained by including all tested variables in a multiple regression models.

| Independent Variables | Coefficient | Standard Error | P Value |
| --- | --- | --- | --- |
| **Constant** | **19.607** |  |  |
| **V1 Upper Limb Fugl-Meyer** | **-0.446** | **0.07** | **0.0001** |
| **SLF I FA Values** | **31.183** | **11.086** | **0.0169** |
| Age | -0129 | 0.106 | 0.26 |
| CST FA Values | 25.029 | 26.346 | 0.3699 |
| SLF II FA Values | 55.799 | 26.698 | 0.07 |

Correlations between FA values in the CST, SLF I and SLF II at V1 and motor recovery, or outcome at V3 were performed using Spearman's rank-order correlations. The results are reported in supplementary Table 3.

**Table 3. Correlations between FA values in the CST, SLF I and SLF II at V1 and motor recovery, or motor outcome at V3**

Correlations were performed using Spearman's rank-order correlations. Values are coefficient [95 % CI]. *: p value < 0.05

| FA Values | MOTOR SCORES | | | |
| --- | --- | --- | --- | --- |
|  | Upper Limb Fugl-Meyer | | Hand Grip Strength ratio | |
|  | Motor Outcome | Motor Recovery | Motor Outcome | Motor Recovery |
| CST | 0.495  [-0.048 ;0.812] | -0.267  [-0.699 ;0.307] | 0.515 *  [0.067 ;0.843] | 0.485  [-0.036 ;0.799] |
| SLF I | -0.328  [-0.731 ;0.245] | 0.502 *  [0.007 ;0.799] | -0.300  [-0.704 ;0.251] | 0.164  [-0.380 ;0.624] |
| SLF II | 0.240  [-0.333 ;0.683] | 0.510 *  [0.197 ;0.803] | -0.054  [-0.551 ;0.472] | -0.371  [-0.742 ;0.174] |

**References**

[1] E. Moulton *et al.*, « Comparison of spatial normalization strategies of diffusion MRI data for studying motor outcome in subacute-chronic and acute stroke », *NeuroImage*, vol. 183, p. 186-199, déc. 2018, doi: 10.1016/j.neuroimage.2018.08.002.

[2] B. Jeurissen, J.-D. Tournier, T. Dhollander, A. Connelly, et J. Sijbers, « Multi-tissue constrained spherical deconvolution for improved analysis of multi-shell diffusion MRI data », *NeuroImage*, vol. 103, p. 411-426, déc. 2014, doi: 10.1016/j.neuroimage.2014.07.061.

[3] J.-D. Tournier *et al.*, « MRtrix3: A fast, flexible and open software framework for medical image processing and visualisation », *NeuroImage*, vol. 202, p. 116137, nov. 2019, doi: 10.1016/j.neuroimage.2019.116137.

[4] R. E. Smith, J.-D. Tournier, F. Calamante, et A. Connelly, « SIFT: Spherical-deconvolution informed filtering of tractograms », *NeuroImage*, vol. 67, p. 298-312, févr. 2013, doi: 10.1016/j.neuroimage.2012.11.049.

[5] K. Rojkova, E. Volle, M. Urbanski, F. Humbert, F. Dell’Acqua, et M. Thiebaut de Schotten, « Atlasing the frontal lobe connections and their variability due to age and education: a spherical deconvolution tractography study », *Brain Struct. Funct.*, vol. 221, n^o^ 3, p. 1751-1766, avr. 2016, doi: 10.1007/s00429-015-1001-3.

[6] M. Catani et M. Thiebautdeschotten, « A diffusion tensor imaging tractography atlas for virtual in vivo dissections », *Cortex*, vol. 44, n^o^ 8, p. 1105-1132, sept. 2008, doi: 10.1016/j.cortex.2008.05.004.

[7] P. J. Winklewski, A. Sabisz, P. Naumczyk, K. Jodzio, E. Szurowska, et A. Szarmach, « Understanding the Physiopathology Behind Axial and Radial Diffusivity Changes-What Do We Know? », *Front. Neurol.*, vol. 9, p. 92, 2018, doi: 10.3389/fneur.2018.00092.

[8] L. Concha, D. W. Gross, B. M. Wheatley, et C. Beaulieu, « Diffusion tensor imaging of time-dependent axonal and myelin degradation after corpus callosotomy in epilepsy patients », *NeuroImage*, vol. 32, n^o^ 3, p. 1090-1099, sept. 2006, doi: 10.1016/j.neuroimage.2006.04.187.
